# Revisiting the vascularity of the keratinized gingiva in the maxillary esthetic zone

**DOI:** 10.1186/s12903-021-01445-y

**Published:** 2021-03-25

**Authors:** Barbara Mikecs, János Vág, Gábor Gerber, Bálint Molnár, Georg Feigl, Arvin Shahbazi

**Affiliations:** 1 Department of Conservative Dentistry, Semmelweis University, Budapest, Hungary; 2 Department of Anatomy, Histology and Embryology, Semmelweis University, Budapest, Hungary; 3 Department of Periodontology, Semmelweis University, Budapest, Hungary; 4 Department of Macroscopic and Clinical Anatomy, Medical University of Graz, Graz, Austria; 5 Institute of Anatomy and Clinical Morphology, Witten/Herdecke University, Witten, Germany

**Keywords:** Periodontal surgery, Keratinized gingiva, Flap, Ischemia, Gender

## Abstract

**Background:**

The active arterial-to-arterial collaterals are a significant factor in the prevention of ischemia and extensive tissue necrosis in the case of arterial blockage of various tissues. The present study investigates the mucogingival vasculature in the maxillary esthetic zone mucosa in human cadavers and functionally evaluates the area, which is supplied by the terminal arterioles, on the individual level.

**Methods:**

In the human cadaver study, macroscopic arterial analyses of the anterior maxillary vestibule in 7 specimens were scrutinized by latex milk injection. The tracks of the mucosal branches in relation to the mucogingival junction were investigated. In the functional study, individual gingival blood flow (GBF) changes were measured by laser speckle contrast imaging (LSCI) in 31 young subjects with healthy gingiva before and during 30-s compressions. This was conducted with a ball-shaped condenser. The data was analyzed by the linear mixed model.

**Results:**

The vertically aligned branches of the superior labial artery (SLA) divided into small, slightly deviating sub-branches near the mucogingival junction. These arteries created collateral plexuses and supplied the attached gingiva. The compression of these branches resulted in ischemia coronally with significant individual variation. The ischemia was either apico-mesial, apico-distal, or straight apical to the compression. A significant correlation was found between the ischemic area and the magnitude of the decrease in GBF (r = 0.81, p < 0.001). In males, 77% of the subjects, and 50% of the female subjects had an ischemic response in either region. The horizontal extension of the ischemic area ranged between 0.26 mm and 8.76 mm. Males had significantly higher baseline GBF and larger ischemia than females. At the base of the papilla, significant restoration of GBF was observed during compression in males, but not in females.

**Conclusion:**

The arcade anastomoses formed by the small arteries in the keratinized gingiva of the upper esthetic zone explain the consequences of vertical incisions. The considerable individual variations in ischemic responses might be the reason for unexpected surgical outcomes in some cases. Furthermore, there is increasing evidence that men have different vascular reactivity and/or regulation of collateral circulation than women, which may affect wound healing.

## Background

Clinical observation findings suggest that flap elevations without vertical incisions favor an accelerated blood supply and uneventful wound healing, consequently improving esthetic outcomes and minimizing the risk of scarring [1–6]. However, in some cases of ridge augmentation or surgical endodontics, the incision can be placed at the mesial border of the flap to avoid cutting through the vessels coming from posterior to anterior [7]. It is also suggested to plan the incision perpendicularly from the mesial- or distal marginal course of the gingiva toward the midline of the papilla and gradually turn parallel to the tooth axis [8]. Prolonged periodontal and implant surgery with a vertical incision on the marginal gingiva and additional excessive local anesthetics with epinephrine result in long-term ischemia in the mucogingival flap [9–11]. However, further data are required regarding the influence of the incision design or wounding on the blood flow or angiogenesis in the anterior maxillary vestibule in humans [12–16].

There is a considerable variation regarding a flap’s survival after surgery [5, 13, 16–20]. This phenomenon could be explained by individual variations in surgical skills, but it may be more rational to consider differences between patients. Previous data suggest [5, 18, 19] the existence of significant variations among subjects in blood flow changes after periodontal surgery. Active arterial-to-arterial or arteriole-to-arteriole collaterals are crucial in the prevention of extensive tissue necrosis in cases of arterial blockage extent of heart failure and stroke [21–23]. The abundance is controlled by genes and is relatively uniform across the tissue within a specific subject [23, 24]. Nevertheless, there is scarce evidence available regarding the abundance, functionality, and individual variations of arterial-to-arterial collaterals in human gingiva. The gingiva and the connective tissue of the maxillary esthetic zone are mainly furnished by tributaries of the superior labial artery (SLA) and the infraorbital artery (IOA) [6]. Based on a corrosion cast of dog’s gingiva [25, 26], the sub-branches of these arteries ramify near the mucogingival junction and subsequently split into the arterioles. The arterioles pass deeply through the connective tissue of the attached gingiva and subdivide into smaller branches, forming a vascular network in the keratinized gingiva [26, 27].

The aims of the present human study are i) to characterize the mucogingival vasculature in maxillary esthetic zone mucosa by utilizing latex milk injection in cadavers and ii) to functionally evaluate the terminal arterioles branching from the vascular network in the keratinized gingiva via laser speckle contrast imaging (LSCI) in healthy volunteers.

## Methods

The investigation is divided into two sections: i) human cadaver study of anterior vestibule arterial analyses in seven maxillae (five dentate, two partially dentate). Seven bodies (four males, three females; 43–69 years of age) donated to science were investigated at the Department of Macroscopic and Clinical Anatomy of the Medical University of Graz, Austria. The bodies were donated in accordance with the Department’s Donation Program and Styrian burial law. Donations were provided exclusively by the person’s own last will and written consent. ii) gingival blood flow (GBF) study involved 31 volunteers (13 males, 18 females) with healthy periodontal statuses. Their ages ranged from 21 to 29 years (mean age: 24). Exclusion criteria were pregnancy, smoking, clinically relevant systemic diseases, and any medication except for contraceptives. The participants granted their written informed consent before they underwent any procedure. The research was performed according to the Declaration of Helsinki (amended in 2013). Ethical permission was granted by the Hungarian authority, called the Committee of the Health Registration and Training Center (approval number: 092642/2015/OTIG).

(i) Latex milk injection in human cadavers

The protocol ensued from the previous study [28]. The common carotid arteries (CCAs) were dissected and canulated. Then, intravascular Thiel solution (to flush the vessels) was introduced to the CCAs [28]. Cautiously, a red-colored agent (Pintasol red E-L3 mix paste, Mixol-products Diebold GmbH, Kirchheim, Germany) was combined with latex milk (Creato Latexmilch, Zitzmann Zentrale, Baden, Germany), and the mixture was injected via the CCAs [6, 28]. The cadavers were embalmed within Thiel solution for about 6–8 months [6, 28]. After that, the path of the arteries was examined.

(ii) Blood flow measurements in healthy volunteers

Subjects were asked to avoid brushing, gargling, rinsing, eating or drinking 60 min before the session. Fifteen minutes before and during blood flow measurements, the patients were comfortably seated and relaxed in a standard supine position on a dental chair in a quiet room with a constant temperature of 24 °C. The patient’s head was fixed by a vacuum pillow. The lips were retracted carefully with a lip retractor (Spandex®, Hager & Werken, Germany). At the beginning, during and end of the intervention, each participant’s blood pressure was measured with an automatic blood pressure meter (Omron M2, Omron Healthcare Inc., Kyoto, Japan) on the left arm. The GBF was evaluated by LSCI (PeriCam PSI HR System, Perimed AB, Stockholm, Sweden). The LSCI illuminates the object with infrared red laser light. The laser light reflected from static objects forms a speckle pattern. However, moving cells blur this pattern proportionally to their velocity [29, 30]. By calculating the contrast of the captured image, LSCI can assess the microcirculation in a non-contact and non-invasive manner with high spatial and temporal resolution. It has an adequate reproducibility in human gingiva [31].

**Fig. 1:**
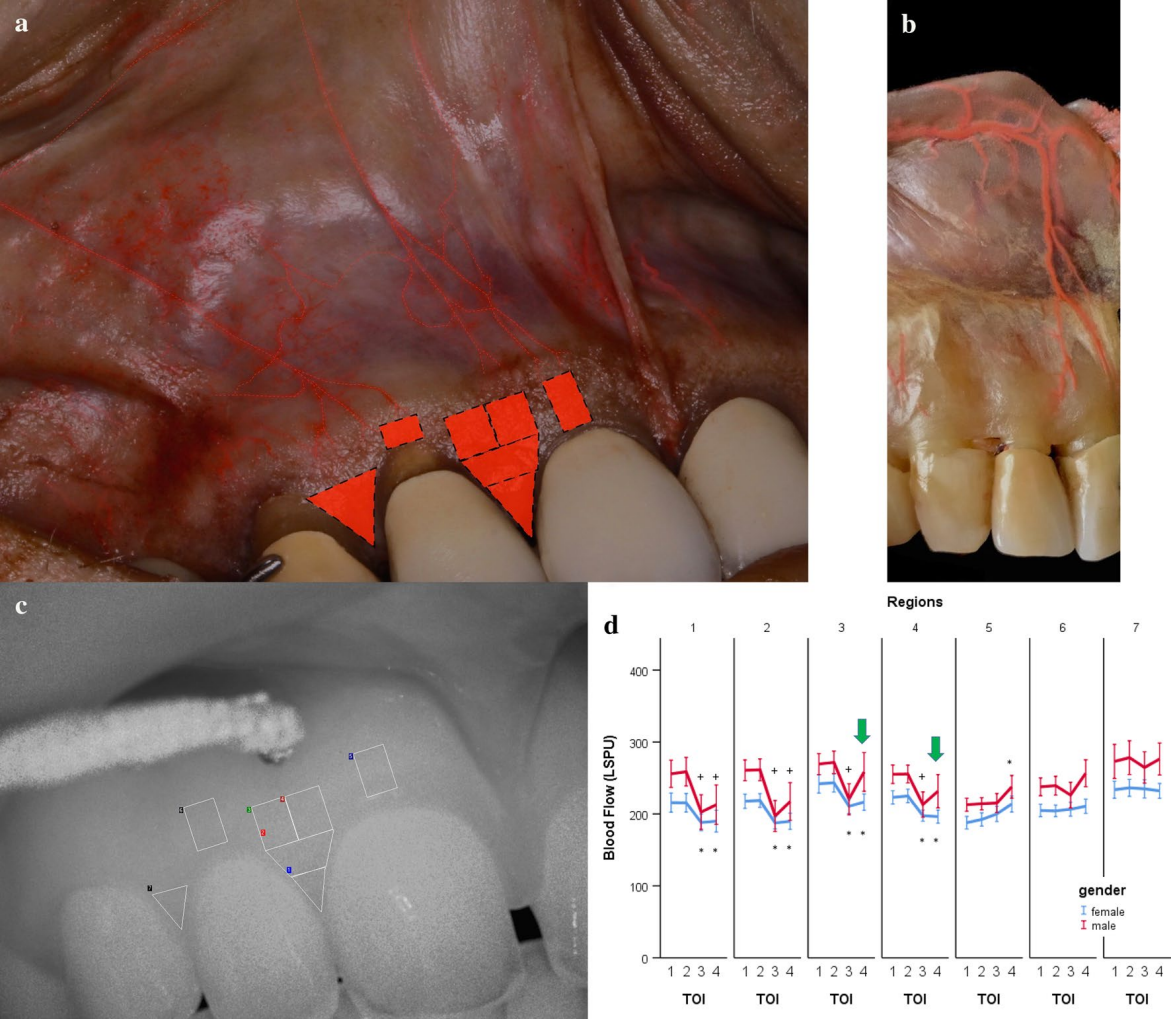
The upper oral vestibule esthetic zone with regions of interest. **a** The superior labial artery (SLA) gives a vertically orientated sub-branches that ramify (near the mucogingival junction) to slightly deviated tributaries moving toward the gingival papillae. The regions analogus to the LSCI’s ROIs are indicated by red rectangules. **b** The vertical branch of the SLA above the right upper incisors, bisects to small arteries and supply the mucosa. **c** Selection of the primary ROIs during the occlusion on the native LSCI image. **d** The mean and the standard error of the blood flow values, expressed in LSPU, in various ROIs in males (red line) and females (blue line). Significant differences compared to the baseline (TOI 1) are indicated by an asterisk, * in males and + in females, p < 0.05 after Bonferroni adjustment. Green arrows indicate a significant rebound in males during the second half of the compression

The region of interest measured approximately 2 × 3 cm; five images/sec were recorded, and they were averaged, thus registering one value per second. The resolution was set to 0.02 mm/pixel. The GBF was expressed in an arbitrary laser speckle perfusion unit (LSPU). After stabilization of GBF, the baseline value was recorded. The compression was made by a ball-headed metal instrument with a diameter of 1.5 mm (Fig. 1c). This procedure was followed by a single investigator that applied a constant force of approximately 20 g for 30 s. If a branch of the vessel was identified above the papilla, then it was selectively compressed. If no distinct vessel was seen, then the middle the most reddish area was compressed.

**Fig. 2:**
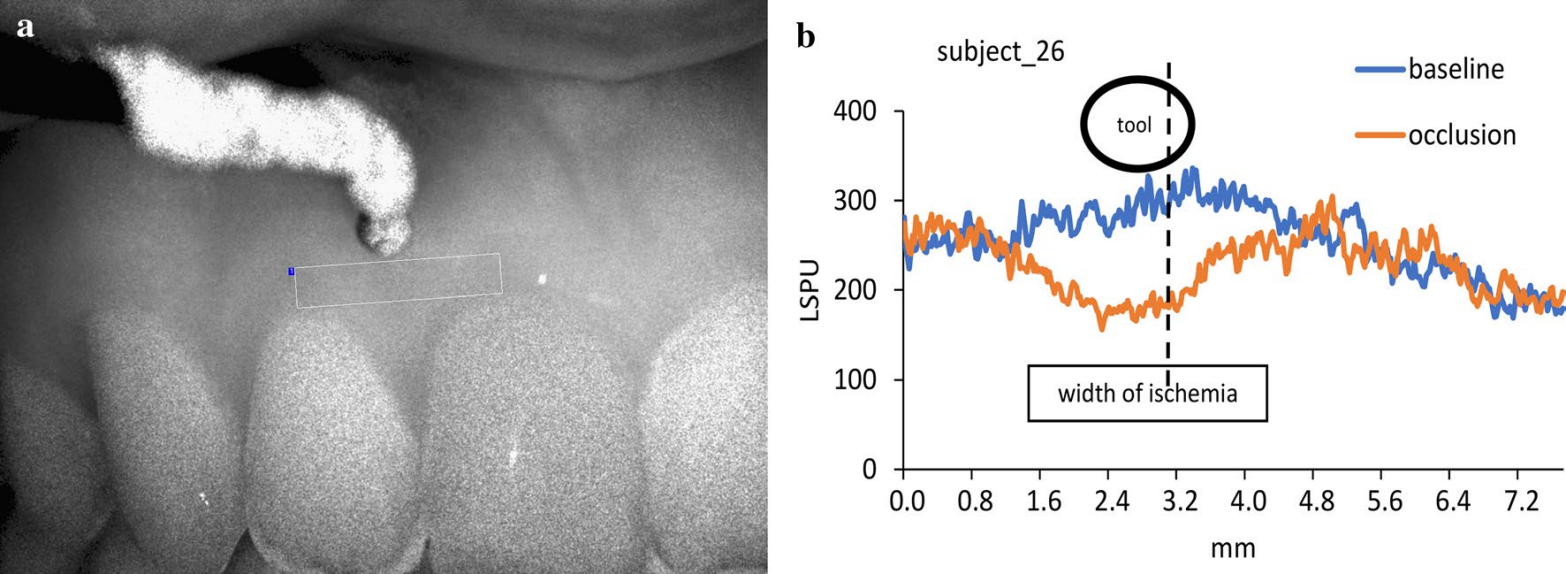
Measurements of the horizontal extension of the ischemia. **a** Selection of ROI 8 for the assessment of horizontal dimension of ischemia. **b** The method of analysis of the raw data. The rectangle indicates the area between the crossing points of the blood flow curves measured at the baseline and under occlusion. The dashed line indicates the midline of the papilla

Primarily, seven regions of interest (ROI) were selected on the native speckle image (Fig. 1c) to obtain an overview of the affected area. ROI 1 and ROI 2 were selected on the papillary area by dividing the height of the papilla into half. ROI 3 and ROI 4 were just above ROI 2 and below the compression tool. ROI 5 and ROI 6 were the same size as ROI 3 and ROI 4 which were placed in the mid-buccal line of teeth 11 and 12. ROI 7 was selected on the papilla between teeth 12 and 13. This had the most significant distance from the compression; therefore, it was expected to be suitable for the reference region. Time of interests (TOI) were selected spanning 10-s intervals. The elapsed time between TOI 1 and TOI 2 (in the baseline period) and between TOI 3 and TOI 4 (in the compression period) was 10 s. Based on the findings of the primary ROIs, the selection of ROI 8 was constructed horizontally, connecting at the mid-buccal point of the teeth 11 and 12 (Fig. 2a).

### Data processing

The means of each primary ROI within each TOI were exported to a Microsoft Excel file. The data were analyzed in multiple steps. First, the LSPUs of all primary ROIs were statistically tested to ensure the stability of the reference ROI 7 and to calculate the mean change within a ROI during vascular occlusion. Then, the reference ROI 7 was selected to calculate the variance component between the TOIs, indicating the variance in time due to the random, within-subject noise. The repeatability coefficient (r) was utilized to determine the smallest difference that indicated a real change (with a 95% confidence interval) at the individual level [20, 31–33]. If the change in any of the 31 cases during occlusion was less then ‘r’ value, then the case was classified as ischemic due to the occlusion.

The ROI 8 was analyzed by exporting every single pixel that contained perfusion values (several thousand each) within the ROI into Excel from the TOI 2 (baseline) and TOI 3 (occlusion). The mean LSPUs for each column (vertical lines) of the horizontal ROI were calculated and graphically depicted (Fig. 2b). In the case of ischemia, the curves of the TOI 2 and TOI 3 formed two crossing-points. The number of pixels between the crossing points were utilized to calculate the horizontal dimension of the ischemic area; the dimension was calculated in mm by multiplying the number of pixels by 0.02 mm. The area under the curve of the TOI 2 was deducted from the area under the curve of TOI 3. This value was divided by number of pixels to calculate the mean magnitude of the ischemia for each case.

### Statistical analysis

Data are presented as mean ± standard error of the mean. Blood flow alterations were investigated with a linear mixed model. The ROIs and TOIs were the main factors, and their interactions were integrated into the model; the respective baseline values were covariates. Pairwise comparisons were made with the least significant difference post hoc test. The p values were adjusted utilizing the sequential Bonferroni method for multiple pairwise comparisons, which was set at 0.05. The relationship between the horizontal dimension of the ischemic area and the mean magnitude of the ischemia was calculated with the Pearson correlation coefficient. The proportion between genders was tested via the Chi-square test. Statistical assessment was made by SPSS 25 (IBM SPSS Statistics for Windows, Version 24.0. Armonk, NY: IBM Corp).

## Results

### Vascular distribution in the maxillary esthetic zone of gingiva

**Fig. 3:**
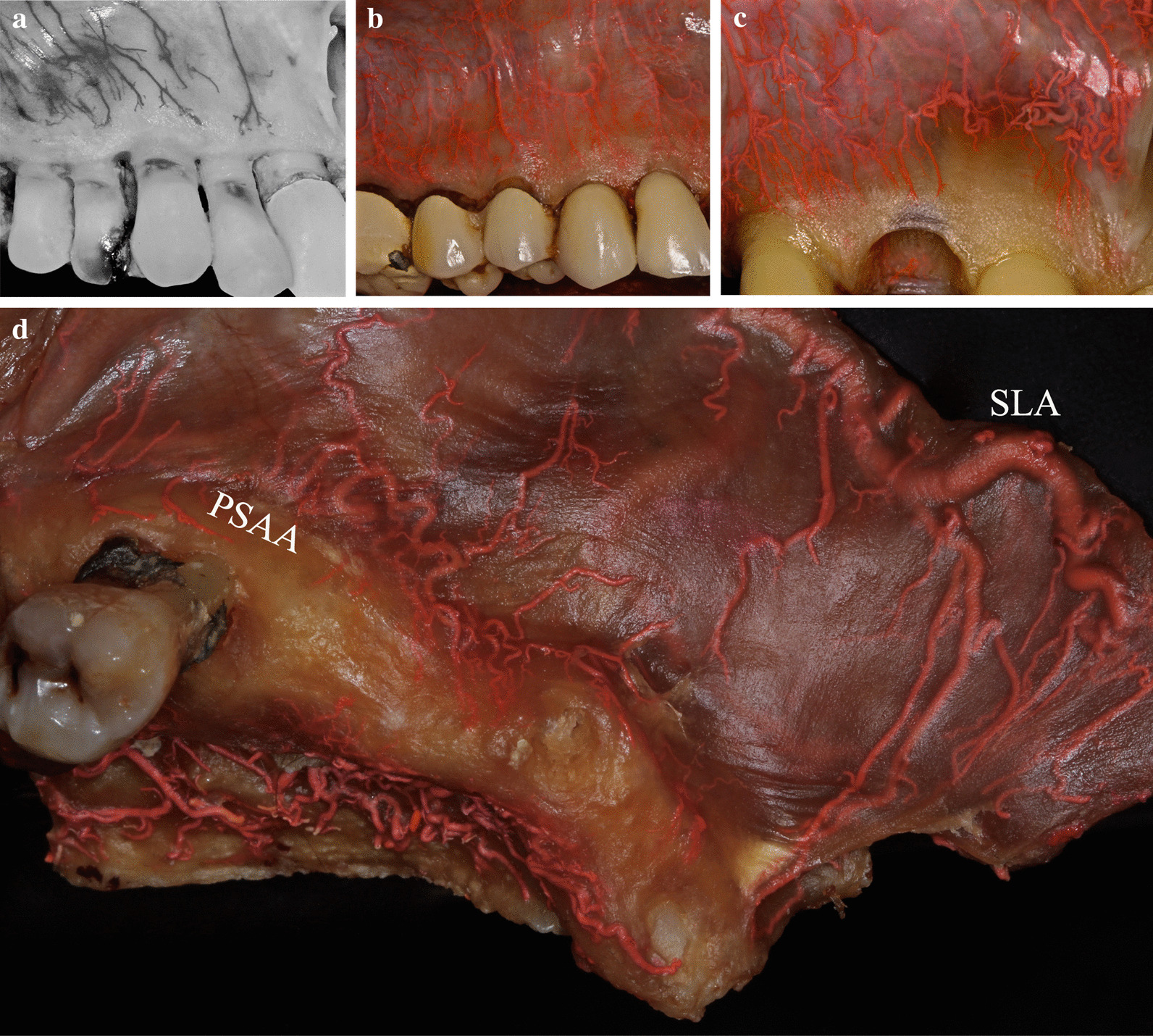
Distribution of the arterial network before and after the maxillary canines in cadavers. **a** The small arteries in the upper right premolar area are mesialized compared to the esthetic zone. **b** The meshwork of anastomoses at the level of the upper right canine, supplies several parts of the gingiva. **c** Magnified small arterial network in the esthetic zone gingiva. **d** Envisioning the tributaries of the posterior superior alveolar artery (PSAA) and superior labial artery (SLA) under the mucosa

At least three vertically positioned arteries originating from the SLA supplied the mucosa of the maxillary esthetic zone (Fig. 1). Following the border of the movable and attached mucosae, these arteries ramified into small, slightly deviating branches (Figs. 1, 3). As the vessels neared the gingival margin and the papilla, the number of arterial mesh increased, and their diameters decreased. According to our observations, the midpoint of the gingiva did not receive an individual branch. Additionally, the pattern of arcade collateral anastomoses above and below the mucogingival junction was observed (Figs. 1, 3). Furthermore, the aligning of the arteries in the posterior and anterior aspects of the maxillary gingiva were compared (Fig. 3a, d). Due to the track of the posterior superior alveolar artery (PSAA), the orientation of the mucosal branches at the premolar zone was more mesialized than the esthetic zone (Fig. 3a, d).

### Effect of compression on the microcirculation of GBF

**Fig. 4:**
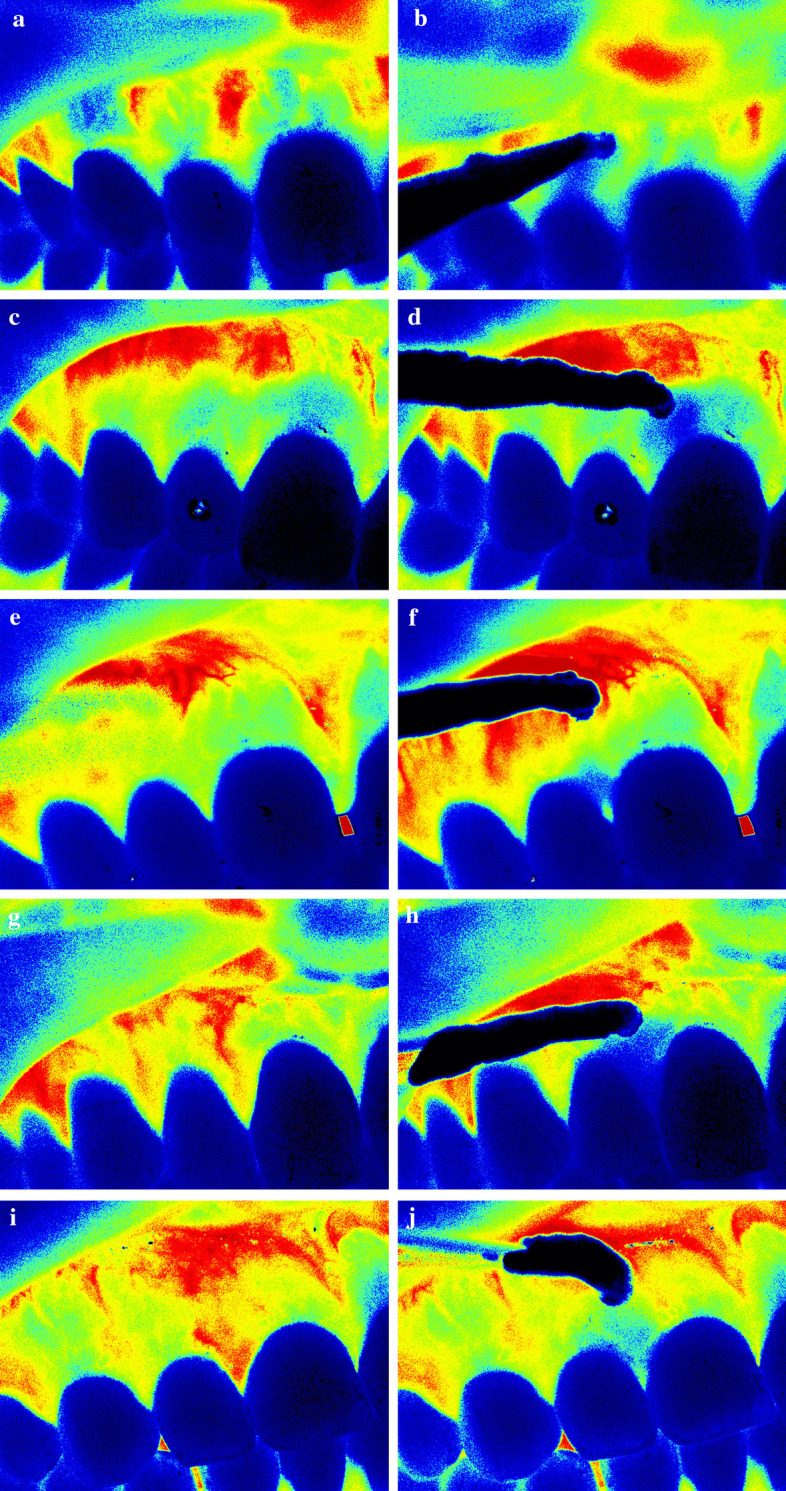
Five cases demonstrate considerable individual variability. **a** In Subject 6, a single vessel can be seen, and **b** ischemia developed in the attached gingiva, distal to the compression with a minor effect on the papilla. **c** In Subject 11, no single vessel can be seen, and **d** ischemia developed in the attached gingiva mesial to the compression with a minor effect on papilla. **e** In Subject 8, a single small vessel was compressed, and **f** only the papilla appears ischemic. **g** In Subject 7, a single large vessel was seen, and **h** a wide, symmetrical ischemic area with complete papillary involvement was developed. **i** In Subject 25, a diffuse reddish area can be seen, and **j** a wide, symmetrical ischemic area with papillary involvement was developed

The vascular pattern of the compressed area was categorized into two groups. In 8 cases (26%), the pattern appeared diffuse (Fig. 4c, i), and in 23 cases (74%), a single vessel could be identified for compression (Fig. 4a, e, g). The center of the compression varied in localization between 1.58 mm distal or 2.20 mm mesial to the midline of the papilla. However, it was always located within the dimension of the papilla base. The change in blood flow after compression was not dependent on the baseline patterns (Fig. 4).

No significant change in GBF was observed between two baseline TOIs (TOI 1 and TOI 2) in any regions (Fig. 1d). Men had significantly higher GBF than women in all regions and all TOIs (Fig. 1d). In females, the GBF of ROI 1, 2, 3, and 4 significantly (p < 0.001–0.05) dropped by -25 to -31 LSPU with similar magnitudes in TOI 3 and TOI 4. In males, the GBF of ROI 1, 2, 3, and 4 significantly (p < 0.001) dropped in TOI 3 by -42 to -64 LSPU. However, the GBF of TOI 4 was significantly (p < 0.001) higher than that of TOI 3 in ROI 3 and ROI 4. The GBF was restored to the baseline in these ROIs during the occlusion. The significant interaction between time and gender was observed during occlusion (TOI 3, p < 0.05), indicating that ischemia was higher in males than in females. In ROI 5 and 6, no ischemic response was observed, but in the ROI 5 at TOI 4, the blood flow increased significantly by 25 LSPU (p < 0.01). In ROI 7, no significant change was observed in any TOIs (+ 1–5 LSPU).

The horizontal extension of the ischemia in the attached gingiva was assessed by analyzing of ROI 8. The mean position of the ischemic area was shifted mesially by 0.27 mm relative to the compression. The shifting varied significantly between subjects; it ranged from 1.51 mm distal to 2.59 mm mesial. The mean width of the ischemic area was 3.8 ± 0.38 mm and ranged between 0.26 mm and 8.76 mm between subjects. The width was not significantly different between genders (3.29 ± 0.46 mm in females, 4.51 ± 0.62 in males, p = 0.116).

The calculated ‘r’ of the reference ROI 7 was 33.3 LSPU. Every change less than -33 LSPU or greater than 33 LSPU was considered a real difference within a specific subject with 95% confidence. In males, 10 subjects of 13 (77%) had an ischemic response in either the papillary or attached gingiva. In females, only 9 of the 18 subjects (50%) indicated an ischemic response, but the difference in proportion between genders was not statistically significant (p = 0.129). The vascular rebound during the 30-s ischemic period (blood flow was elevated by at least 33 LSPU from the TOI 3 to 4) was seen in 8 (38%) of the ischemic cases. The incidence of the rebound was not significantly different between genders (5 males and 3 females). The distribution of biotypes between genders was not different (thin-scalloped: 6 females and 5 males, thick-flat: 7 females and 3 males, thick-scalloped: 5 females and 5 males; p = 0.635). No differences in blood flow were observed between biotypes.

**Fig. 5:**
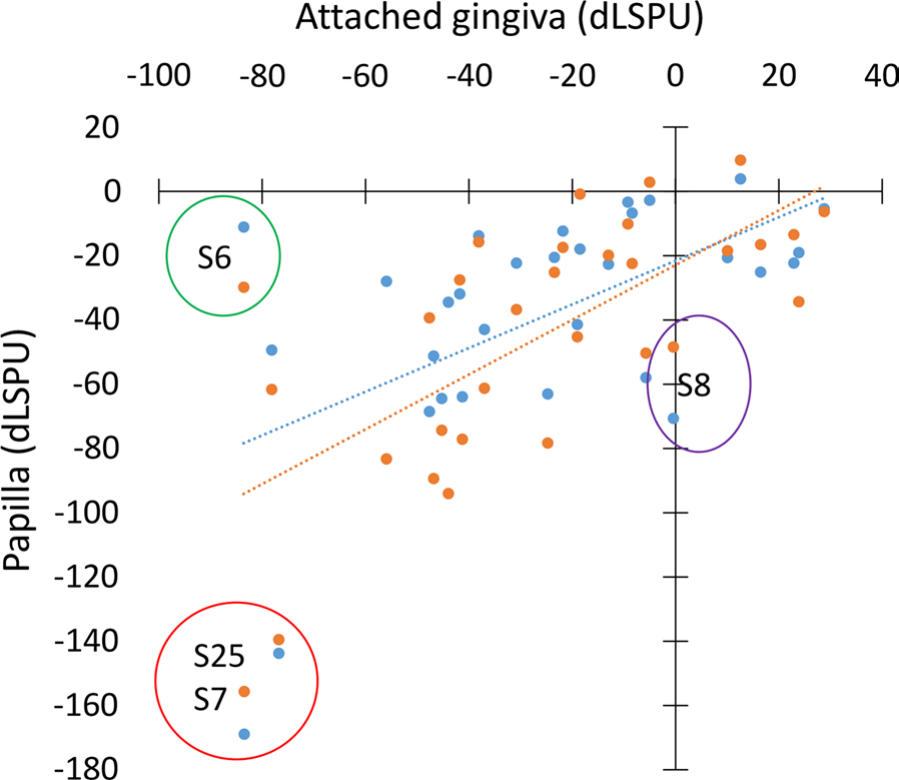
Correlation between the change in blood flow in the attached gingiva (ROI 8) and papilla (ROI 1 and 2). ROI 1 (blue points) is localized at the papilla tip; ROI 2 (orange points) is localized at the base of the papilla. The blue and orange dots are usually close together. Dashed lines indicate the linear regression between attached gingiva and, ROI 1 (blue) and ROI 2 (orange). A green circle indicates a subject with significant ischemia in the attached gingiva, but lack of significant ischemia in the papilla. The purple circle indicates papillary ischemia. Red circles indicate two subjects with extremely high ischemia in all three ROIs

A moderate correlation in the magnitude of ischemia was observed between the attached gingiva (ROI 8) and the papilla (r = 0.56, p < 0.01 for ROI 1 and r = 0.68, p < 0.001 for ROI 2; Fig. 5). There was a strong correlation between the magnitude of the horizontal extent of the ischemia in the attached gingiva (ROI 8) and the mean drop in blood flow (r = 0.81, p < 0.001).

## Discussion

During incision and flap design, acknowledgement of vascular track and decent circulation results in proper angiogenesis, vasculogenesis, and primary wound healing [7, 34, 35]. The maxillary vestibule presents a complex vascular circulation [6], which might be the cause of intra- and postoperative complications. Specifically, the movable mucosa of the maxillary esthetic zone receives at least three vertical branches originating from the SLA. According to our observations, the arteries were approximately localized above the upper front teeth. The high-resolution image captured by the LSCI indicated that there is a zone above the papilla which has higher microcirculation, and it is in accordance with previous findings [20, 36, 37]. In cadavers, no sign of a rich arterial network was discerned in the mid-papillary area compared to the mid-buccal area. Therefore, the differences could be explained by the level of the blood flow regulation and imply more intense microcirculation of the interdental area.

Previously [15, 16, 20], it has been emphasized that the arterioles in the attached gingiva are positioned primarily vertically. Similarly, Shahbazi et al. [6] have proposed that the sub-branches of the SLA (mainly supplying the mucosa/gingiva) and IOA (mainly supplying the periosteum/gingiva) in the upper esthetic zone have vertical orientations. Latex milk injection in both earlier [6] and current experiments reveal that following the junction of the movable and attached mucosae, the vertical arteries bisect to smaller branches. These diminutive sub-branches became slightly deviated and moved toward different parts of the attached gingiva. Similarly, the direction of the ischemic (mesial, distal oblique, or straight vertical) pattern after compression of the terminal branch varied among patients. Cadaver specimens also indicated a collateral anastomoses arcade network above and below the mucogingival junction in the upper esthetic zone. In periodontal and implant-related surgeries, predominantly, the arcade anastomoses in the attached mucosa are significant in the circulation of the gingiva [6]. During secondary wound healing of the exposed periosteum, the revascularization primarily begins laterally [5], which emphasizes the significance of horizontal branches. The study [5] also suggested limiting the horizontal extension of the surgical wound. Therefore, to maintain proper circulation and prevent ischemia in the keratinized gingiva, it is suggested to avoid vertical incisions in the marginal gingiva [5, 38]. The scar tissue formation directly correlates with the tissue hypoxia and delayed revascularization [5, 38, 39]. This coincides with the root coverage study [3] stating vertical incision can let in keloid formation.

In the cadaver investigation, number and information regarding the clinical condition of the gingivae were limited. Furthermore, previous studies indicated that vascular resistance, microcirculation pulse curve, and vascular morphology tend to change with the age [40–42]. Therefore, we aimed to functionally confirm the heterogeneity among patients by studing only young subjects with healthy gingivae. A previous study [20] applied a 10 mm wide horizontal compression mid-buccally. During the compression, no blood flow was distinguished in any of the cases, indicating a complete closure of the blood supply within the affected area. Some individuals in the present study experienced little or no ischemia after 1.5 mm wide compression. Therefore, the size of the area without effective compensation can be confined between 1.5 and 10 mm. This is in the range of the average distance of the vessels that supplied the attached gingiva observed in cadavers; however, it does not explain the individual variations. Generally, the arteriole-to-arteriole anastomoses interconnect a small part (generally < 0.05%) of arterioles in the crowns of neighboring arterial trees [43]. Their size on average is less than 100 μm in diameter in the majority of healthy species (including humans), and can be detected in most of the tissues [39]. The arteriole-to-arteriole anastomosis was rarely observed in the attached gingiva in a recent study [27]. This could explain the reason that some of our patients had extensive ischemia horizontally and vertically, although only a 1.5 mm wide area was blocked. Furthermore, these subjects also had the highest ischemia in magnitude. The subjects may have a single large vessel supplying a large area, up to 8 mm, without any existing collaterals. The size of vessels in the attached gingiva are less than 100 µm, according to the previous literature [25, 44], which indicates that the whole attached gingiva belongs to the microcirculation network. Conversely, large arteries (> 200 µm) in the deep layer of the attached gingiva have recently been revealed [27]. We suppose that in some instances the large artery may move directly toward the papilla without branching to attached gingiva, explaining the outcome in which only the papilla was ischemic. In this study, only 61% of the volunteers presented ischemia in different directions. The lack of response could be explained by the small sub-branches may run parallel (not visible in cadavers), and they could bypass the occluded branch. By acknowledging individual variations, surgical complications and failure might be prevented.

The blood vessels of the interdental papilla arise from interdental septa, periodontal ligament, and the gingiva [45]. The significant contribution of the gingiva in maintaining the papillary blood flow has recently been demonstrated [20]. The complete compression of the papilla base resulted in a considerable drop in the blood flow of the papilla. In the present study, although only a 1.5 mm area at the mucogingival line was occluded, in many cases, we found significant ischemia in the papilla. These results suggest that in physiological conditions, the main blood supply of the papilla originate from gingival vessels. During incision/flap designs, such as papilla preservation flap [10] or horizontal incision [5], the previously described alveolar and periodontal supplements may become activated by a mechanism called collateralization [43].

In addition to the suggested cross-connection between branches in the attached gingiva, collaterals between supraperiosteal and periodontal plexuses were elucidated in histological studies in dogs and cats [25, 44]. Conversely, the wide horizontal compression of the attached gingiva in humans demonstrateed no collateral circulation from the periodontal ligament [20]. Wounding the attached gingiva by 1.5 mm diameter punching either mid-buccal or mid-papillary [15] revealed seven days of ischemia coronal to the wound, which confirmed the significance of the supply from the vestibule. The study [15] measured 2.06 mm of the horizontal width of the ischemic area six hours after wounding. Notably, in our investigation, we applied a similar localization and dimension for a reversible blockade. We found that the mean horizontal width of the ischemic area was double in size (3.8 mm) than in the wounding study. The difference in extent might be explained by vasodilation and by collateralization within the six hours of healing. The relative standard deviation (i.e., coefficient of variation) of the whole ischemic area in the earlier wound study [15] resembles our findings (70%), similarly indicating significant individual variations. They [15] also observed the quicker revascularization of the mid-papillary area than the mid-buccal and confirmed the higher vascular perfusion of the mid-papillary zone.

The arteriovenous bundles run together when they enter keratinized gingiva; they branch and form a microcirculation mesh [25, 26]. The simultaneous blockade of venular outflow could prevent the refill of the vessels from the collaterals. However, in some cases, a significant rebound could be observed with a considerable variation in anastomosis mesh between individuals. In this study, males were found to be more prone to compensate the ischemia by opening collaterals. It has been previously found that males experience higher hyperemia responses after a complete closure of vessels in the attached gingiva [20]. Furthermore, the circulation of the coronally advanced flap was restored much earlier in males than in females [18], and there is evidence that males have better wound healing than females after a third molar surgery [46–49] as well as after palatal wounding [50]. Better blood flow recovery may facilitate wound healing in males. In previous investigations [5, 51, 52], blood flow changes induced either by nitric oxide, epinephrine, or surgery were not correlated with the gingival thickness measured by the ultrasonic instrument. Therefore, gender differences cannot be explained by biotype. A higher magnitude of ischemia in males indicates a higher vascular reactivity; however, the similar extent of the ischemic area in males and females suggests a similar distribution of the vessels.

## Conclusion

Generally, the main arteries originating in the vestibule supply the attached gingiva by providing small, slightly deviated branches. This even distribution does not explain the considerable heterogeneity of the resting blood flow of the attached gingiva; thus, the regulation of the microcirculation may vary between sites. Furthermore, the localization and responsiveness of the collaterals considerably depend on the individual and the genders.

## Data Availability

The datasets utilized and analyzed during the study.

## Abbreviations

LSCI: Laser Speckle Contrast Imaging
SLA: Superior labial artery
IOA: Infraorbital artery
PSAA: Posterior superior alveolar artery
GBF: Gingival blood flow
CCAs: Common carotid arteries
LSPU: Laser Speckle Perfusion Units
ROI: Regions of interest
TOI: Time of interests
